# Management of Adults With Bacterial Meningitis in the Emergency Department

**DOI:** 10.7759/cureus.62767

**Published:** 2024-06-20

**Authors:** Joshua Asemota, Iulia Stoian, Godson Amaze, Saheed Olayinka, Noel Uchenna, Mandar Marathe

**Affiliations:** 1 Internal Medicine, Leicester Royal Infirmary, Leicester, GBR; 2 Anaesthesia, Royal Cornwall Hospitals NHS Trust, Truro, GBR; 3 General Practice, Royal Cornwall Hospitals NHS Trust, Truro, GBR; 4 General Practice, Mersey and West Lancashire Teaching Hospitals NHS Trust, Prescot, GBR; 5 Emergency Medicine, Leicester Royal Infirmary, Leicester, GBR

**Keywords:** critical medication, meningitis, healthcare, quality improvement, emergency department

## Abstract

Introduction: The Leicester Royal Infirmary Emergency Department is one of the largest single-site Emergency Departments in the UK. We evaluated the department’s management of bacterial meningitis. The current national guideline recommends that all patients presenting with suspected bacterial meningitis receive antibiotics within one hour.

Methods: A survey of 100 clinicians (Consultants, Registrars, House Officers, and Advanced Clinical Practitioners) working in the Emergency Department was performed to determine the awareness of the guidelines and a retrospective examination of case notes for patients who presented at the Leicester Royal Infirmary Emergency Department with suspected meningitis was carried out between May 1, 2022, and May 1, 2023. A random sample of 30 patients was drawn from the department's database of 190 patients, identified through discharge coding summaries.

Results: Nine (25%) of the prescribers knew of the guidelines for managing meningitis, and six (16.7%) had utilised the hospital guidelines. Thirty-three (91.7%) prescribers acknowledged the importance of administering steroids to patients suspected of having bacterial meningitis (excluding those displaying signs of meningococcal sepsis, such as a rash). However, only seven (23%) of patients received this treatment. Additionally, only one (3.3%) patient was documented as having received a dose within the first hour of presentation.

Conclusion: The timely diagnosis and administration of appropriate antibiotic therapy are pivotal elements in managing bacterial meningitis. As a result, we designed a checklist to facilitate the effective management of meningitis within the department by increasing awareness of the guidelines and making the critical principles of suspected meningitis management more accessible.

## Introduction

Bacterial meningitis constitutes a medical emergency [1]. Despite the availability of effective and suitable antibiotics, bacterial meningitis in adults remains a challenging disease marked by high mortality and morbidity [2]. This condition can be life-threatening if not suspected, appropriately diagnosed, and promptly managed [3]. Delays in management may stem from late arrival at a medical institution or delays in instituting appropriate management after the patient has presented to an emergency department [4]. Clinicians must recognise the importance of prioritising initial investigations while not delaying life-saving treatment with antibiotics and dexamethasone (if meningococcal meningitis is not suspected). This should be given promptly within the first hour following presumptive diagnosis [1].

Bacterial meningitis was often deadly from its recognition in 1805 into the early 20th century [5]. The first meningitis outbreak was recorded in Geneva in 1805 [6]. Gaspard Vieusseuax and Andre Matthey in Geneva and Elisa North in Massachusetts first described epidemic meningitis [6]. Austrian bacteriologist Anton Vayselbaum was the first to link bacterial infection as a cause of meningitis [6]. The incidence of Meningococcus among household contacts is about 5%. According to estimates, the risk of developing meningitis after being exposed to a patient with meningococcal meningitis is 500 to 800 times greater than in the general population [7]. In the past decades, there have been significant changes in the epidemiology of bacterial meningitis, mainly because of the introduction of conjugate vaccines targeting prevalent meningeal pathogens such as Meningococcal, Pneumococcal and Haemophilus influenzae serotype b (Hib) vaccines [3]. The prevalence of meningitis has consistently decreased over the last two decades [8]. The incidence of bacterial meningitis was 0.9 per 100,000 persons annually in the U.S. in 2014, less than half the rate in the 1970s and 1980s [8]. Emergency clinicians are, however, still likely to encounter cases of bacterial meningitis despite the decreasing incidence, with an estimated prevalence of meningitis among ED visits at 62 per 100,000 [8]. Bacterial meningitis can be fatal if untreated, with reported mortality rates of 30% for pneumococcal and 10% for meningococcal infections [8]. Emergency clinicians play a crucial role in diagnosing and managing meningitis, as delays can lead to significant morbidity and result in higher healthcare costs for the patient [8].

The suspicion of meningitis arises from the manifestation of suggestive symptoms, including fever, nuchal rigidity, headache and altered mental status [4]. The differential diagnosis of bacterial meningitis encompasses the causes of aseptic meningitis, which include infectious (primarily viral but also partially treated bacterial meningitis and focal central nervous system infections) and non-infectious causes, including neoplasm, drugs, and systemic diseases [7]. The primary causative agents of community-acquired bacterial meningitis include Streptococcus pneumoniae, Neisseria meningitides and Haemophilus influenza type B. Globally, bacterial meningitis is associated with cooler, drier seasons [9]. Climate change will likely impact the incidence of meningitis, but modelling data remain inconclusive [10]. Additionally, social distancing measures introduced to curb the spread of SARS-CoV2 during the coronavirus infectious disease 2019 pandemic are predicted to result in a 20 - 30% reduction in the incidence of meningitis [11].

The role of adjunctive dexamethasone therapy in patients with bacterial meningitis must be considered. The rationale for the use of dexamethasone is based on findings from experimental animal models of infection, revealing that the subarachnoid space inflammatory response with bacterial meningitis plays a significant role in contributing to morbidity and mortality. Diminishing this inflammatory response may be effectual in reducing the various pathophysiologic outcomes of bacterial meningitis, such as cerebral oedema, elevated intracranial pressure, altered cerebral blood flow, cerebral vasculitis, and neuronal injury; complications which are mediated by expression of proinflammatory cytokines [12-14].

In community-acquired meningitis, selecting the most effective initial empirical antibiotic regimen depends on the regional resistance patterns. The UK Joint Specialist Societies guideline on the diagnosis and management of acute meningitis and meningococcal sepsis in immunocompetent adults recommends that all patients with suspected meningitis or meningococcal sepsis in the community be referred to a hospital for evaluation, urgent antibiotic administration due to the rapid progression and severe nature of the disease and consideration of lumbar puncture (LP). This one-hour window is crucial because early treatment improves outcomes significantly, reduces the risk of severe complications, and increases the chances of survival [15].

Prior studies showed low adherence to national guidelines for meningitis management [16,17]. Based on existing data, this study assessed prescribers' awareness of the national guidelines and evaluated their adherence.

## Materials and methods

This study used a clinician survey and a retrospective review of the case notes of patients treated for meningitis in the Emergency Department.

Table 1 shows the questionnaire sent to 100 Emergency Department clinicians. This was performed to assess their awareness of the guideline. The survey was designed with closed-ended questions with nominal variable responses. It was conducted virtually by distributing a Google Form (Google LLC, Mountain View, USA) via a broadcast email to Emergency Medicine Clinicians of all grades (Consultants, Registrars, House Officers and Advanced Clinical Practitioners) at Leicester Royal Infirmary who prescribe medications. Therefore, a voluntary sampling method was used to obtain responses. The survey received 36 responses (36% response rate), which were included in the analysis. 

**Table 1:** Meningitis management questionnaire

No.	Questions
1	Are you aware of any meningitis pathway available at the University Hospitals of Leicester?
2	Have you ever used the pathway called “*Bacterial meningitis and meningococcal septicaemia in adults”*?
3	Where would you look if you were searching for this pathway?
4	What is the first-line treatment for meningitis according to the guidelines?
5	Besides antibiotics, which other intravenous supportive treatment should be used?
6	What investigations would you request in the Emergency Department for a patient with suspected meningitis?

Case note review

For the retrospective review of case notes, the study population included patients aged 18 and above diagnosed with suspected or confirmed meningitis in the Emergency Department from the hospital’s electronic database. The study's time frame focused on patients who presented between May 1, 2022 and May 1, 2023.

We used a simple random sampling method to review 30 patients from the 190 patients who presented during this timeframe. Each patient’s care was audited against the following national guidelines: (i) All immunocompetent adults aged 16-60 should be given ceftriaxone 2g IV with empirical dexamethasone within one hour of hospital presentation except if allergic, clinical suspicion of meningococcal sepsis or the medication was already prescribed in the community and (ii) add amoxicillin if the patient is at risk of Listeria monocytogenes (60 years or older or immunocompromised)

The questionnaire was analysed using Google Docs, while the retrospective case study results were analysed using Microsoft Excel (Microsoft Corporation, Redmond, USA). Patient-identifiable and clinician-identifiable data were handled in accordance with the institution's information governance procedures. Responses and patient identifiers were anonymised. Institutional approval was obtained for the whole project. 

## Results

Table 2 shows the results of the questionnaire administered to clinicians, with the number of correct responses and percentages out of the 36 total responses.

**Table 2:** Results of the questionnaire with the number (%) of correct responses

No.	Questions	Number of correct responses (percentage)
1	Are you aware of any meningitis pathway available at the University Hospitals of Leicester?	9 (25%)
2	Have you ever used the pathway called “*Bacterial meningitis and meningococcal septicaemia *in adults”?	6 (16.7%)
3	Where would you look if you were searching for this pathway?	21 (58.3%)
4	What is the first-line treatment for meningitis according to the guidelines?	27 (75%)
5	Besides antibiotics, which other intravenous supportive treatment should be used?	33 (91.7%)
6	What investigations would you request in the Emergency Department for a patient with suspected meningitis?	22 (61.1%)

Adherence to antibiotic administration

**Figure 1:**
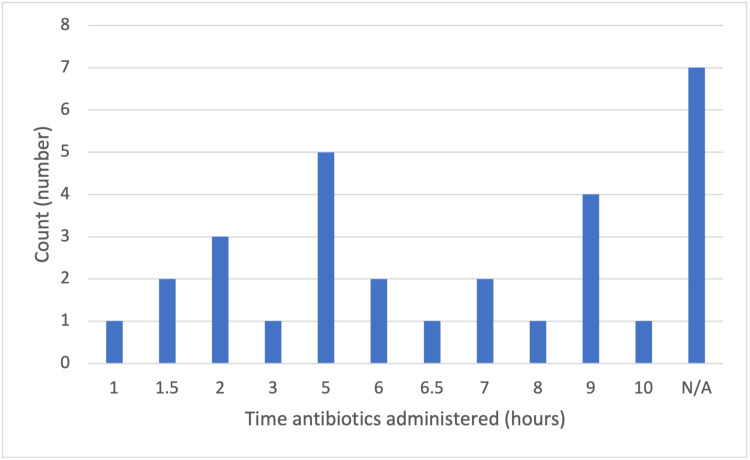
Time antibiotics administered from presentation

Figure 1 depicts the time patients received the antibiotics from the time of presentation to the hospital (Mean 5.4, SD 2.8). Although the delay in administration is associated with poorer outcomes, the overall neurological state of the patient at the time of antibiotic initiation correlates more with the outcome. Various techniques have been attempted to reduce delays in patient management, such as a study to assess the effect of prompt LPs in patients (including those with impaired mental status, reducing time spent obtaining a CT head and awaiting its report). The study concluded that prompt LPs lead to an overall shorter time until antibiotics are administered and more favourable patient outcomes [18]. 

**Figure 2:**
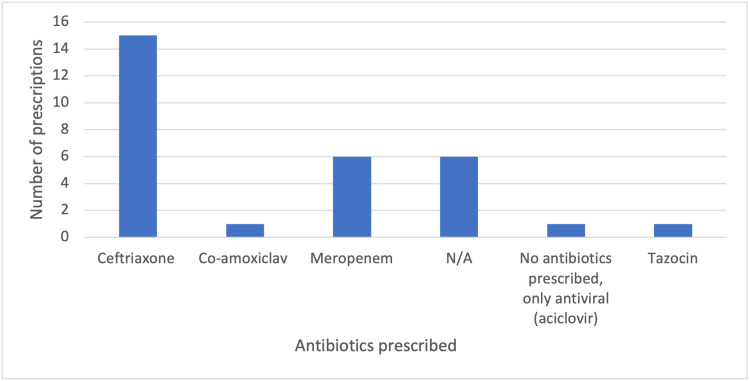
Type of antibiotics prescribed

Figure 2 shows the type of antibiotics given. Fifteen (50%) patients presenting to the Emergency Department received the appropriate first-choice antibiotic, ceftriaxone.

Adherence to additional recommendations

Table 3 shows the percentage of immunocompromised patients who received amoxicillin. Six patients were identified as at risk of listeria due to being immunocompromised, and only one (16.7%) received amoxicillin to cover the probable listeria infection. 

**Table 3:** Percentage of immunocompromised patients who received amoxicillin

Amoxicillin given	Count of immunocompromised patients	Percentage
No	5	83.3%
Yes	1	16.7%

Table 4 shows the percentage of patients who received steroids, with only seven (23.3%) patients receiving steroids with antibiotics.

**Table 4:** Percentage of patients who received steroids

Count of steroid given	Steroid given	
Pt pregnant /above 60 years/immunocompromised (including diabetics and alcohol abuse)	No	Yes
No	18	6
Yes	5	1
Total (percentage)	23 (76.7%)	7 (23.3%)

After reviewing case notes, it was observed that out of the patients admitted with suspected meningitis, seven (23.3%) received a final diagnosis of meningitis after LP. 

## Discussion

Our study retrospectively examined patient cases at the Emergency Department of a tertiary care centre in the United Kingdom between May 1, 2022, and May 1, 2023. It sought to evaluate the concordance between clinical practices and established guidelines for managing suspected bacterial meningitis and meningococcal septicaemia in adults, as outlined by the national guidelines.

Timely management of patients with bacterial meningitis is necessary to prevent adverse outcomes. Although the national target for administering antibiotics is one hour, this is not routinely met [19] and is getting longer due to the increased pressures on the emergency systems. This delay in an emergency occurs for numerous reasons, for example, for patients requiring cerebral imaging before LP can be performed. Although guidelines strongly recommend that antibiotics should still be prescribed within one hour of presentation, even if an LP and imaging cannot be completed within the time frame, non-adherence by clinicians leads to this delay [4]. There is an association between the delayed duration of antibiotics administration from the time of presentation and worse patient outcomes [20].

Our study showed that only nine (25%) individuals surveyed were aware of the specific guideline designed to guide the management of bacterial meningitis, and six (16.7%) had used the guideline. The result of the survey also showed that approximately half of the clinicians had the correct idea of where to access hospital guidelines and knew the complete investigations to request as per the national guidelines (21(58.3%) and 22(61.1%), respectively). These findings indicate gaps in disseminating information among healthcare providers, which are unveiled in the varying perceptions regarding the primary treatment for meningitis in a tertiary centre, with a considerable number of respondents (9 (25%)) failing to identify ceftriaxone 2g + amoxicillin as the recommended first-line treatment and only 15 (50%) of patients received the recommended antibiotic, ceftriaxone, showcasing a significant discrepancy between the ideal course of action outlined in the guidelines and the actual administration in clinical settings. This signifies clinicians' need for more awareness of the optimal treatment protocol. The result also sheds light on the discrepancies in adherence, particularly in high-risk patient populations, including immunocompromised, elderly, and pregnant individuals, showing a low rate of patients at risk of listeria receiving IV Amoxicillin (Table 3: 1 (16.7%)).

Steroids should also be administered in addition to antibiotics, especially if pneumococcal meningitis is suspected and there are no contraindications to steroid administration, such as clinical evidence of meningococcal septicaemia (a non-blanching purpuric rash). Steroids have been found to reduce the incidence of hearing loss and neurological sequelae associated with meningitis management [21]. From the survey responses, the majority of the clinicians (33 (91.7%)) are aware that steroids should be administered to patients with suspected meningitis (except for patients who have signs of meningococcal sepsis, such as a rash). However, only seven (23%) patients received the treatment, suggesting potential gaps in implementing specific treatment protocols.

Although the delay in administration is associated with poorer outcomes, the overall neurological state of the patient at the time of antibiotic initiation correlates more with the outcome. Various techniques have been attempted to reduce delays in patient management, such as a study to assess the effect of prompt LPs in patients (including those with impaired mental status, reducing time spent obtaining a CT head and awaiting its report). The study concluded that prompt LPs lead to an overall shorter time until antibiotics are administered and more favourable patient outcomes [18]. From a review of patients' notes, only one (3.3%) patient was documented to have received antibiotics within one hour of presentation to the Emergency Department. Bacterial meningitis is a relatively rare life-threatening disease, and physicians accustomed to managing meningitis, such as infectious disease specialists, had a significantly earlier time to administer antibiotics from patient presentation than non-infectious disease specialists [22]. This is partly due to the early identification of the disease by specialised clinicians and initiation of treatment, not delaying it for investigations such as CT head or LP, which leads to significantly better overall outcomes [23]. Hands-on experience in managing meningitis, instead of solely relying on theoretical knowledge, results in better overall outcomes. This is evident in a study which emphasised that infectious disease doctors, compared to non-infectious disease doctors, administered steroids and the initial antibiotic dose to a significantly higher number of patients. This issue is addressed in the latest national guidelines that advocate that patients with suspected bacterial meningitis should be managed by a senior clinician with competencies in caring for acutely ill patients [24].

Due to various diseases like viral upper respiratory tract infections and sinusitis that can imitate meningitis and are more prevalent, the Emergency Department’s diagnosis of meningitis has a low positive predictive value as most cases of meningitis diagnoses in the Emergency Department are based on clinical presentation, which has a high sensitivity with low specificity. Hence, patients with suspected meningitis should undergo LP irrespective of findings from physical examination alone, as it is insufficient to confirm or exclude meningitis definitively [25]. A comparative study conducted at an urban tertiary centre revealed that the Emergency Department team achieved an accuracy rate of 28.9% in diagnosing meningitis. This determination was made by comparing patients admitted with suspected meningitis to the final diagnosis upon discharge, following additional investigations [26]. This finding aligns with the seven (23%) patients who received a final diagnosis of meningitis on discharge. This relatively low percentage of meningitis patients following extensive workups underlines the complexity involved in accurately diagnosing these conditions, raising questions about the reliability of initial clinical assessments and highlighting the need for more precise diagnostic methods.

The complications resulting from the disease include neurological and non-neurological ones. Outcomes are excellent with adequate antibiotic treatment and other supportive therapies. In our study, mortality was one (3.3%) patient who died from complications of comorbidities. Factors affecting mortality include age, long duration of symptoms before seeking medical attention, lack of neck stiffness and obtunded mental state on admission [27].

**Figure 3:**
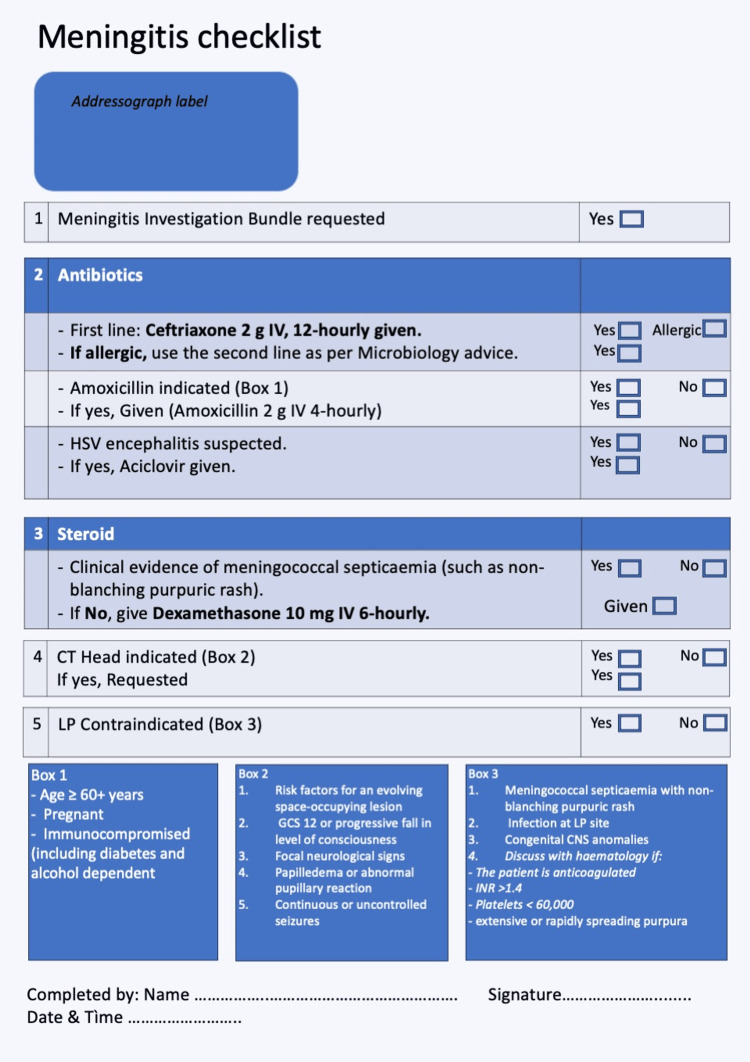
Meningitis management checklist CNS: Central nervous system; CT: computed tomography; g: gram; GCS: Glasgow Coma Scale; HSV: Herpes Simplex Virus; INR: International Normalised Ratio; IV: intravenous; LP: lumbar puncture; mg: milligram

Our study underscores substantial gaps between established guidelines and clinical practices in managing suspected bacterial meningitis and meningococcal septicaemia cases at a tertiary centre, possibly due to a lack of awareness and guidance. The findings emphasise the critical need for heightened awareness, comprehensive education and rigorous adherence to established protocols among healthcare providers. Addressing these gaps is imperative to ensure optimal patient care, accurate diagnoses and improved clinical outcomes for individuals with these life-threatening conditions. To solve this, we designed a meningitis checklist highlighting the critical elements of investigations and management for patients presenting to the front door of the Emergency Department with suspected meningitis to be completed by the admitting clinician (see Appendices, Figure 3). We also created an informative poster to raise awareness in the department and an investigation and treatment bundle on the electronic prescription and medicine administration software to aid clinicians and prevent delayed/missed investigation and treatment.

Study limitation

The study focuses on managing suspected bacterial meningitis in the Emergency Department. As viral (and other causes) cannot be excluded without LP, we advise giving antiviral medication in combination with antibiotics if symptoms of viral encephalitis are present. This study involved a relatively small sample size of thirty patients for the case note review. The voluntary sampling method for participants' responses to the questionnaire could introduce selection bias towards individuals interested or more aware of the topic.

## Conclusions

Identifying a patient with meningitis early in the disease course and administering the appropriate treatment has been shown to improve patient outcomes in terms of morbidity and mortality, which led to the development of a one-hour national target for treating all patients presenting with suspected bacterial meningitis. Several factors contribute to delays and substandard management, including the human factor (clinicians needing more proficiency in diagnosing the disease or having limited experience with meningitis management), time consumed by procedures like LPs, imaging and waiting for results. Our study showed clinicians' low awareness of the guidelines, which is reflected in patient management as highlighted in the case note review. To address this issue, we designed an information poster, checklist, investigation and treatment bundle to assist clinicians in efficiently managing suspected bacterial meningitis patients. Further evaluation in subsequent studies would assess the intervention's impact on ongoing patient management.
